# Runs of Homozygosity Do Not Influence Survival to Old Age

**DOI:** 10.1371/journal.pone.0022580

**Published:** 2011-07-25

**Authors:** Maris Kuningas, Ruth McQuillan, James F. Wilson, Albert Hofman, Cornelia M. van Duijn, André G. Uitterlinden, Henning Tiemeier

**Affiliations:** 1 Department of Epidemiology, Erasmus University Medical Center, Rotterdam, The Netherlands; 2 Centre for Population Health Sciences, University of Edinburgh, Edinburgh, United Kingdom; 3 Netherlands Genomics Initiative (NGI)-sponsored Netherlands Consortium for Healthy Aging (NCHA), Leiden, The Netherlands; 4 Department of Internal Medicine, Erasmus University Medical Center, Rotterdam, The Netherlands; 5 Department of Child and Adolescent Psychiatry, Erasmus Medical Center – Sophia Children's Hospital, Rotterdam, The Netherlands; Ohio State University Medical Center, United States of America

## Abstract

Runs of homozygosity (ROH) are extended tracts of adjacent homozygous single nucleotide polymorphisms (SNPs) that are more common in unrelated individuals than previously thought. It has been proposed that estimating ROH on a genome-wide level, by making use of the genome-wide single nucleotide polymorphism (SNP) data, will enable to indentify recessive variants underlying complex traits. Here, we examined ROH larger than 1.5 Mb individually and in combination for association with survival in 5974 participants of the Rotterdam Study. In addition, we assessed the role of overall homozygosity, expressed as a percentage of the autosomal genome that is in ROH longer than 1.5 Mb, on survival during a mean follow-up period of 12 years. None of these measures of homozygosity was associated with survival to old age.

## Introduction

Runs of homozygosity (ROH) are extended tracts of adjacent homozygous single nucleotide polymorphisms (SNPs) that are more common in unrelated individuals than previously thought. Recent evidence has shown that in European populations all individuals have runs of homozygosity (ROH) shorter than 1.5 Mb, reflecting ancient LD patterns or the chance inheritance of common haplotypes from both parents, whereas larger ROH reflect recent parental relatedness or selective advantage [1], [2], [3]. These large ROHs are likely to be enriched for loci harbouring functional variants.

To date, a number of studies have analyzed genome-wide SNP data for ROH and identified individual ROHs that increase the risk of schizophrenia [1] or Alzheimer's disease [4], and associate with height [5] in unrelated individuals. However, for bipolar disorder [6], colorectal cancer [7] and childhood acute lymphoblastic leukemia [8] no associations with individual ROH have been found. Furthermore, no study has reported associations with overall measures of homozygosity. As for human longevity, it is likely that recessive alleles that promote longer life exist but have so far not been identified with genome-wide association studies (GWAS). Making use of the ROH approach might help to identify loci harbouring these alleles.

In this study, we examined ROH larger than 1.5 Mb individually and in combination for association with survival in 5974 participants of the Rotterdam Study. In addition, we assessed the role of overall homozygosity on survival during a mean follow-up period of 12 years. None of these measures of homozygosity was associated with survival to old age.

## Results and Discussion

In order to assess the role of ROH in human longevity and to identify novel longevity loci, we examined overall homozygosity, and individual ROH regions in the whole genome that are larger than 1.5 Mb. For this purpose, we made use of the genome-wide genotype data available in the first and second cohort of the Rotterdam Study (RS) [9] (**Table 1**). After quality control 530132 SNPs in 5974 participants were available for subsequent analyses in the RS1 discovery cohort. Genome-wide ROH were identified using the Runs of Homozygosity program implemented in PLINK software [10]. We defined ROH as runs of at least 25 consecutive homozygous SNPs spanning more than 1.5 Mb with a maximum gap between two SNPs 100 Kb and a minimum SNP density coverage of at least 20 SNPs per Kb. As a measure of overall homozygosity and level of inbreeding, we calculated a percentage of the autosomal genome that is in ROH longer than 1.5 Mb [2].

**Table 1:** Characteristics of the Rotterdam Study (RS).

	RS1	RS2
	(Discovery cohort)	(Replication cohort)
N	5974	1895
Female (n, %)	3547 (59%)	1032 (55%)
Age at baseline in years (mean, SD)	69.4 (9.10)	64.8 (8.03)
Follow-up in years (mean, SD)	12.4 (5.19)	7.62 (1.55)
Mortality (n, %)	3174 (53%)	242 (13%)

Altogether 0.44% (standard deviation (SD) 0.31) of the genome was in ROH longer than 1.5 Mb in the 5974 participants of RS1. This percentage is comparable to that reported for other outbred populations [2]. Of the 5974 participants, 3174 (53%) died during a mean follow-up period of 12 years (**Table 1**). When assessing the association of genome-wide homozygosity with survival, we observed no statistically significant association (hazard ratio (HR), 95% confidence interval (CI); 0.95, 0.85–1.07). Even though the estimate is below unity, suggesting that higher genome-wide homozygosity is beneficial for survival to old age, the effect size is too small to be of any relevance.

Next, we assessed the association between individual ROH regions and survival in RS1. For this analysis all overlapping ROHs within a region were merged, yielding 1040 regions that had a frequency higher than 1%. Of these regions nine were associated with survival in RS1 (**Table 2**). To validate the existence of these ROH regions and their association with survival, we made use of the genome-wide SNP data in 1895 participants of RS2 (**Table 1**). We detected the same ROH regions also in RS2, but with different frequencies. Furthermore, none of these associated with survival in RS2 (**Table 2**). For most estimates the direction was opposite to that observed in RS1, suggesting that the initial findings arose due to chance. However, the lack of replication could also stem from the smaller sample size, shorter follow-up and lower number of events in the RS2 cohort.

**Table 2:** Association between survival and individual ROH regions.

				RS1		RS2
	Length	SNP	Freq		Freq	
ROH region	(KB)	(N)	(%)	HR (95% CI)	(%)	HR (95% CI)
chr8:68132797–86055273	17923	3313	1.19	1.73 (1.29–2.33)	2.90	0.59 (0.22–1.59)
chr3:49499240–55909341	6410	969	9.73	1.21 (1.08–1.36)	10.6	1.02 (0.66–1.57)
chr3:164109279–170486328	6377	838	1.14	1.49 (1.11–2.00)	2.16	0.77 (0.29–2.06)
chr1:168915903–194976780	26061	3984	7.13	1.17 (1.02–1.35)	10.5	1.12 (0.74–1.70)
chr6:139849739–157503677	17654	3905	1.10	0.66 (0.47–0.94)	3.43	0.92 (0.47–1.80)
chr2:40481216–70916156	30435	6603	1.54	1.34 (1.04–1.72)	4.80	1.00 (0.56–1.78)
chr12:75054501–94547494	19493	3230	4.10	1.20 (1.00–1.43)	7.60	1.14 (0.73–1.79)
chr8:86915545–113724772	26809	4584	1.57	0.73 (0.55–0.98)	13.9	1.18 (0.82–1.69)
chr23:80214069–88330836	8117	610	1.04	1.38 (1.01–1.88)	1.69	1.81 (0.74–4.45)

Cox proportional hazard regression adjusted for sex and age at baseline; *p<0.05; Freq-frequency; HR-hazard ratio; CI-confidence interval.

To determine if survival to old age is influenced by the combination of several ROHs we used the total number of ROH per individual variable provided in the PLINK output. This measure takes also infrequent ROHs into account. Altogether 5957 (99.7%) of the participants carried one or more ROH longer than 1.5 Mb with a maximum of 29 different ROHs per individual (mean 5.83 ROH, SD 2.62). Despite the inclusion of infrequent ROH, we observed no association between the number of ROH per individual and survival (HR, 95% CI; 0.99, 0.98–1.01).

The lack of associations observed in this study could lie in the definition of ROH. In this study we used the criteria proposed by McQuillan R *et. al.* [2], whereas in other studies different criteria have been used [4], [5], [6], [7], [8]. The most commonly varying criteria is the length of ROH. In this study we concentrated on ROH larger than 1.5 Mb, but in other studies ROH larger than 1.0 Mb [4] or 0.5 Mb [5] have been analysed. In the latter study, also different minimum number of SNPs constituting a ROH, and SNP density coverage were used. To determine whether the results of our study would change with different ROH definition criteria, we repeated the analyses using ROH larger than 1.0 Mb and 0.5 Mb, and setting the number of SNPs constituting a ROH and SNP density to that used in the study of Yang TL *et. al*.[5]. However, also with these definitions of ROH, we did not observe statistically significant associations between genome-wide homozygosity and survival (data not shown).

In conclusion, we found no association between survival and overall homozygosity, and we could not identify ROH regions that influence survival to old age either individually or cumulatively. Despite the lack of statistically significant associations, these results do not exclude the existence of recessive loci that affect survival to old age. However, it is likely that these loci have small effects and occur at low frequencies in the general population.

## Materials and Methods

### Study population

The Medical Ethics Committee of the Erasmus Medical Center approved the study and written informed consent was obtained from all participants. The Rotterdam Study (RS) is an ongoing population-based cohort study on risk factors for chronic diseases in the elderly. Detailed information on design, objectives and methods has been presented elsewhere [9]. For this study data from the first (RS1) and second (RS2) cohort of the Rotterdam Study were available. In RS1, all inhabitants aged over 55 years living in the Ommoord district of Rotterdam were invited to participate. Of these 7983 (78%) agreed to participate. In 1999, 3011 participants (out of 4472 invitees) who had become 55 years of age or moved into the study district since the start of the study and were added to the initial cohort (RS2). All participants of the Rotterdam Study were followed for mortality until January 1 2009. The current study included 5974 participants of the RS1 and 1895 participants of RS2.

### Genotyping

The genome-wide genotyping was performed with Illumina 550K array (Illumina, San Diego, CA, USA) in self-reported individuals of European descent (sample call rate ≥97.5%). Individuals with excess of autosomal heterozygosity, mismatch between genotypic and phenotypic gender, and outliers identified by the identity-by-state (IBS) clustering analysis were excluded. In addition, individuals with more than 5% missing genotypes were excluded. SNPs with a call rate less than 90%, minor allele frequency (MAF) lower than 1%, or failing Hardy-Weinberg equilibrium (HWE) at a threshold of p<0.0001 were discarded, leaving 530132 SNPs for subsequent analyses.

### Identification of runs of homozygosity (ROHs)

Genome-wide ROHs were identified using the Runs of Homozygosity program implemented in PLINK v1.07 software [10]. PLINK uses a sliding window of minimum 50 SNPs across the genome to identify ROHs, allowing for five missing SNPs and one heterozygous site per window. We identified ROHs, which were at least 1.5 Mb of consecutive homozygous genotypic calls at adjacent SNP loci, to exclude very common ROHs that occur in all individuals in all populations [2]. The minimum number of continuous homozygous SNPs constituting a ROH was set to 25 and the minimum SNP density coverage was set to at least 20 SNPs per Kb, allowing for centromeric and SNP-poor regions to be algorithmically excluded from analysis. The maximum gap between two consecutive homozygous SNPs was set at 100 Kb. Overall homozygosity was calculated by summing the lengths of all ROH longer than 1.5 Mb and expressing this as a% of the typed autosomal genome. The “homozyg-group” option in PLINK was used to create a file of overlapping ROH regions for each individual.

### Statistical analysis

The association between survival and overall homozygosity and individual ROHs at particular genomic locations and total number of ROH per individual was conducted by Cox proportional hazard regression, adjusted for age at baseline and sex. All association analyses were performed with SPSS version 17 (SPSS Inc, Chicago, IL, USA) statistical software.
